# Acute cerebellitis in children: an eleven year retrospective multicentric study in Italy

**DOI:** 10.1186/s13052-017-0370-z

**Published:** 2017-06-12

**Authors:** Laura Lancella, Susanna Esposito, Maria Luisa Galli, Elena Bozzola, Valeria Labalestra, Elena Boccuzzi, Andrzej Krzysztofiak, Laura Cursi, Guido Castelli Gattinara, Nadia Mirante, Danilo Buonsenso, Claudia Tagliabue, Luca Castellazzi, Carlotta Montagnani, Chiara Tersigni, Piero Valentini, Michele Capozza, Davide Pata, Maria Di Gangi, Piera Dones, Silvia Garazzino, Luca Baroero, Alberto Verrotti, Maria Luisa Melzi, Michele Sacco, Michele Germano, Filippo Greco, Elena Uga, Giovanni Crichiutti, Alberto Villani

**Affiliations:** 1 Pediatric and Infectious Diseases Unit, IRCCS Bambino Gesù Children Hospital, Rome, Italy; 2 Pediatric Highly Intensive Care Unit, Department of Pathophysiology and Transplantation, Universita degli Studi di Milano, IRCCS Ca’ Granda Ospedale Foundation, Policlinico Maggiore, Milan, Italy; 3 Department of Paediatrics, Pediatric Infectious Disease Unit, University of Florence, Meyer Children’s Hospital, Florence, Italy; 4 Pediatric Infectious Diseases Unit, Department of Pediatrics, Catholic University A, Gemelli Hospital, Rome, Italy; 5 Infectious Disease Section, Palermo-Civico Hospital, Azienda di Rilievo Nazionale ad Alta Specializzazione (ARNAS), Palermo, Italy; 6 Department of Paediatrics, University of Turin, Regina Margherita Children’s Hospital, Turin, Italy; 7 Department of Pediatrics, University of L’Aquila, L’Aquila, Italy; 8 Department of Pediatrics, Bicocca, Fondazione MBBM, San Gerardo Hospital, Monza, Italy; 9 Maternal and Pediatric Department, IRCCS CSS Hospital, San Giovanni Rotondo, Foggia Italy; 10 Unit of Clinical Pediatrics, Vittorio Emanuele Hospital, University of Catania, Catania, Italy; 11 Pediatric Unit, ASL Vercelli, Vercelli, Italy; 12 Pediatric Unit, University-Hospital of Udine, Udine, Italy

**Keywords:** Cerebellitis, Children, Outcome, Therapy

## Abstract

**Background:**

Acute cerebellitis (AC) and acute cerebellar ataxia (ACA) are the principal causes of acute cerebellar dysfunction in childhood. Nevertheless. there is no accepted consensus regarding the best management of children with AC/ACA: the aim of the study is both to assess clinical, neuroimaging and electrophysiologic features of children with AC/ACA and to evaluate the correlation between clinical parameters, therapy and outcome.

**Methods:**

A multicentric retrospective study was conducted on children ≤ 18 years old admitted to 12 Italian paediatric hospitals for AC/ACA from 01/01/2003 to 31/12/2013. A score based on both cerebellar and extracerebellar signs/symptoms was computed for each patient. One point was given for each sign/symptom reported. Severity was divided in three classes: low, moderate, severe.

**Results:**

A total of 124 children were included in the study. Of these, 118 children received a final diagnosis of ACA and 6 of AC. The most characteristic finding of AC/ACA was a broad-based gait disturbance. Other common symptoms included balance disturbances, slurred speech, vomiting, headache and fever. Neurological sequelae were reported in 6 cases (5%) There was no correlation among symptoms, cerebrospinal fluid findings, clinical outcome. There was no correlation between clinical manifestations and clinical score on admission and length of hospital stay, sex, age and EEG findings with sequelae (*P* > 0.05).

Children with pathological magnetic resonance imaging (MRI) or computed tomography (CT) had a higher probability of having clinical sequelae.

Treatment was decided independently case by case. Patients with a higher clinical score on admission had a higher probability of receiving intravenous steroids.

**Conclusions:**

We confirmed the literature data about the benign course of AC/ACA in most cases but we also highlighted a considerable rate of patients with neurological sequelae (5%). Pathological MRI or CT findings at admission correlate to neurological sequelae. These findings suggest the indication to perform an instrumental evaluation in all patients with AC/ACA at admission to identify those at higher risk of neurological outcome. These patients may benefit from a more aggressive therapeutic strategy and should have a closer follow-up. Randomized controlled trials are needed to confirm these observations. The ultimate goal of these studies could be to develop a standardized protocol on AC/ACA. The MRI/CT data, associated with the clinical manifestations, may allow us to define the class risk of patients for a neurological outcome.

## Background

Acute cerebellitis (AC) is an inflammatory syndrome characterized by acute onset of cerebellar signs/symptoms (such as ataxia, nystagmus or dysmetria) often accompanied by fever, nausea, headache, altered mental status and brain magnetic resonance imaging (MRI) abnormalities of the cerebellum [1–3]. The absence of an altered mental status and/or MRI abnormalities is the element that differs AC and acute cerebellar ataxia (ACA), which is also known in current literature as post infectious cerebellar ataxia. Because of similarities in the clinical presentation, ACA and AC are regarded by some authors as a continuum with similar pathogenesis [3].

AC/ACA constitutes one of the most important causes of acute cerebellar dysfunction in childhood [4] and is a common reason of pediatric emergency room attendance. AC/ACA is an inflammatory syndrome caused by direct infection or postinfectious autoimmune mechanisms [5, 6].

AC/ACA is associated with a complete recovery in most cases [7], although few reports with severe outcomes have been described [5, 7–9].

There is no accepted consensus regarding the best management of children with AC/ACA [10–12]. Children with moderate and severe AC/ACA are frequently treated with steroids in addition to antivirals in case of suspected viral etiology (either Varicella Zoster Virus (VZV) or Human Herpes Virus-6 (HHV-6)), but there are no studies evaluating both the best management for these conditions and the impact of different therapeutic approaches on the outcome [8].

For this reason, we carried out this multicentric retrospective study aimed to assess clinical, neuroimaging and electrophysiologic features of children with AC/ACA and to evaluate the correlation between clinical parameters and different therapeutic approaches with the outcome.

## Methods

A multicentric retrospective study was conducted collecting data from all children ≤ 18 years old admitted to 12 Italian paediatric hospitals for AC/ACA from 01/01/2003 to 31/12/2013 (Table 1).

**Table 1:** Italian participant centers

Institution	Number of Cases	Location in Italy
Bambino Gesù Children Hospital	51	Rome, Center Italy
Meyer Children University Hospital	12	Florence, Center Italy
IRCCS Ca Grande Hospital Foundation	11	Milan, North Italy
Arnas Hospital	11	Palermo, South Italy
Catholic University	10	Rome, Center Italy
Perugia University Hospital	6	Perugia, Center Italy
Turin University Hospital	6	Turin, Center Italy
San Gerardo Hospital	6	Monza, North Italy
IRCCS San Giovanni Rotondo	5	San Giovanni Rotondo, South Italy
Vittorio Emanuele Hospital	4	Catania, South Italy
ASL	1	Vercelli, North Italy
Hospital of Udine	1	Udine, North Italy

**Fig. 1:**
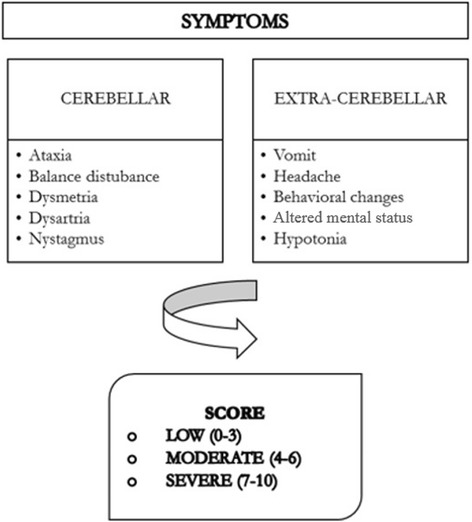
The score proposed to classify AC/ACA severity

Cases were identified by reviewing hospital charts of all children admitted for signs or symptoms indicative of acute cerebellitis. The inclusion criteria were patients with ataxia with/without other cerebellar/extracerebellar symptoms like dysmetria, dysarthria, intention tremor, nystagmus, vomiting, fever, headache, altered mental status or hypotonia. In each case, the following data were collected: sex, age, symptoms at disease manifestations, length of hospital stay, symptoms at discharge, and information about treatment. Acute sickness due to cerebellar symptoms can mimic behavioural changes, but this was assessed only by the clinician not on a subjective parental account. Symptoms at disease manifestation were divided into cerebellar symptoms and extracerebellar symptoms as in Fig. 1. Acute sickness due to cerebellar symptoms can mimic behavioural changes, but this was assessed only by the clinician not on a subjective parental account.

The coordinating center (Bambino Gesù Children Hospital) submitted to all participant centers an online format. Demographic, clinical, laboratory and microbiological data, neurologic investigations, radiologic studies and treatments, outcome and instrumental findings at follow-up were registered.

A score based on both cerebellar (ataxia, dysmetria, balance disturbances, nystagmus, dysartria) and extracerebellar (headache, vomit, hypotonia, behavioral changes, sensory changes) signs/symptoms was computed for each patient. One point was given for each sign/symptom reported. Severity was divided in three classes (Fig. 1). This score was based on a previous, unpublished local experience on over 100 patients with AC/ACA.

Due to the lack of clear guidelines, the use of neuroimaging, as well as the use of steroids/antivirals, was based on the local experience and local protocols.

Follow-up was performed according to the local experience of each center. Follow-up included clinical, neurological and cognitive evaluation; neuroimaging was performed when the local clinician considered it worthwile.

Patients older than 18 years of age, with serious underlying diseases (i.e. congenital or acquired immunodeficiency, chronic renal disease, chronic liver disease, thalassaemia and infantile cerebral palsy) or neurological diseases or malformations, as well as patients who presented with ataxia due to toxic ingestion or tumors were excluded.

### Statistical Analyses

Categorical data were given as number of cases and percentages, continuous variables were reported as median and range.

Chi-square test or Fisher exact test were performed to assess association between categorical variables, while Mann-Whitney test were used for quantitative variables.

A *p* value ≤ 0.05 was considered as statistically significant.

All analysis were carried out using the Stata package for Windows (Stata Statistical Software: Release 14).

### Ethics statement

The study was performed in accordance with the Declaration of Helsinki and approved by the review board of the Italian Pediatric Infectious Diseases Society (Protocol 24/02/2014).

## Results

### Patients’ characteristics

A total of 124 children were included in the study (55% males). Out of them, 118 children received a final diagnosis of ACA and 6 a final diagnosis of AC. The mean age of patients affected by AC/ACA was 5.3 years (SD: ±3.3). VZV infection was diagnosed in 66 cases (53%). Ten children (8%) were VZV immunized and developed AC/ACA not VZV-related. Table 1 summarizes main patient characteristics.

### Characteristics of patients with AC

Six children, 5 boys and 1 girl, aged 2.5 to 9 years, received a diagnosis of AC. Their clinical, laboratory, imaging, and treatment data are summarized in Tables 2 and 3. Routine laboratory findings were normal in all children. Clinical presentation was similar in all cases except cases 1 and 2, who presented headache and behavioral changes in addition to their cerebellar symptoms. These were the only two children who underwent a computed tomography (CT) scan with pathological findings and the only two children with neurological sequelae.

**Table 2:** Patients characteristics and outcome

	With Sequelae		Without Sequelae		*P*.Value
	N	%	N	%	
Sex^a^					
Male	5	83	69	58.4	0.39
Female	1	17	49	41.6	
Age, years^b^ *[median, range]*	4 (3–18)		4 (1–17)		0.41
Score^b^					
Low	2	33	58	49	0.11
Moderate	2	33	53	45	
Severe	2	33	7	6	
Steroids (*N*=61)^a^					
No	3	50	31	56	1
Yes	3	50	24	44	
Antivirals (*N*=71)^a^					
No	3	50	16	25	0.33
Yes	3	50	49	75	
CSF findings (pathological) (*N*=20)^a^					
No	2	50	10	63	1
Yes	2	50	6	37	
MRI (pathological) (*N*=45)^a^					
No	3	50	36	92	0.02
Yes	3	50	3	8	
CT (pathological) (*N*=22)^a^					
No	0	0	20	100	0.004
Yes	2	100	0	0	
EEG (pathological) (*N*=40)^a^					
No	1	50	19	50	1
Yes	1	50	19	50	

*CSF* Cerebrospinal fluid

^a^Fisher exact test

^b^Mann-Whitney test

**Table 3:** Clinical presentation and microbiological results of the 6 children with AC

Case	Age (years), Sex	Aetiological diagnosis	Clinical findings
1	3.5 Male	Blood IgG and IgM positive for coxsackie virus	Ataxia, dysmetria, vertigo, dizziness, hypotonia, headache, behavioural changes, somnolence,
2	4 Male	VZV (clinical diagnosis)	Ataxia, dysmetria, balance disturbances, tremors, nystagmus, dizziness, dysarthria, hypotonia, headache, vomiting, somnolence, behavioural changes
3	6.5 Male	VZV (clinical diagnosis)	Ataxia, fever, vomiting, rigor, somnolence,
4	9 Male	*	Ataxia, dysmetria, balance disturbances, tremors, vomiting, hypotonia,
5	2.5 Male	VZV (clinical diagnosis)	Ataxia, vertigo, tremors
6	8 Female	*	Ataxia, vertigo

*no microbiological findings

### Clinical symptoms and management of patients

As shown in Tables 4 and 5 the most characteristic finding of AC/ACA was a broad-based gait disturbance that gradually progressed over the course of the first 2 days recovery (92%). Other common symptoms included balance disturbances (68%), slurred speech (27%), vomiting (42%), headache (30%) and fever (34%). Poor coordination of finger-to-nose movements (dysmetria) was observed in 35% of cases. In a few cases, nystagmus (12%) was noted. Tone, deep tendon reflexes and plantar responses were normal in all patients.

**Table 4:** CSF, neuroimaging, EEG findings, management and outcome of the 6 children with AC

Case	CSF	Radiology	EEG	Therapy	Outcome
1	Cells 49/mmc, Protein 528 mg/l	RM: Cerebellar leptomeningeal enhancement; CT: area of bilateral cerebellar hypodensity	normal	Aciclovir iv 9 days + 2 weeks Methylprednisolone ev 3 days, then prednisone 1 month IVIg 1 day	Speech disturbances
2	Cells 105/mmc	RM: Cerebellar leptomeningeal enhancement; CT: mild cerebellar edema	Abnormal: diffuse slow activity	Aciclovir iv 14 days Dexamethasone iv 14 days	Ataxia
3	Normal	MRI: abnormal TR signal of cerebellar bulbus and pedunculus	Abnormal: diffuse slow activity	Aciclovir iv 5 days + per os 7 days Dexamethasone iv 4 days + prednisone po 9 days	No sequelae
4	Normal	MRI: Cerebellar leptomeningeal enhancement;	Abnormal: diffuse slow activity	Aciclovir iv 7 days	No sequelae
5	Not done	MRI: mild hyperintensity of cerebellar pedunculus	Not done	Aciclovir iv 5 days	No sequelae
6	Normal	MRI: hyperintensity of cerebellar white matter	Not done	Prednisone po, 20 days	No sequelae

**Table 5:** Clinical cerebellar and extracerebellar symptoms (*N*=124)

	Number	Percent
Cerebellar signs/symptoms		
Ataxia^a^	114	92
Balance distrurbances^b^	84	68
Dysmetria	43	35
Dysartria	34	27
Intentional tremors	30	24
Vertigo^c^	22	18
Nystagmus	15	12
dysdiadochokinesia	9	7
Extracerebellar signs/symptoms		
Vomit	52	42
Fever	42	34
Headache	37	30
Hypotonia	21	17
Altered mental status	3	2.4
Other	12	10

^a^lack of voluntary coordination of muscle movements that includes gait abnormality. ^b^If you are standing, sitting, or lying down, you might feel as if you are moving, spinning, or floating. If you are walking, you might suddenly feel as if you are tipping over. ^c^A brief, intense episode of vertigo triggered by a specific change in the position of the head. ^b^ and ^c^: Definitions by the National Institute of Deafnessand Other Communication Disorders (NIDCD)

Everyone had at least one cerebellar symptom. Eight patients (6%) reported at least one cerebellar symptom and none extracerebellar symptom, while 92% of the cases had at least one cerebellar and at least one extracerebellar symptom. There was no correlation between the number of cerebellar and/or extracerebellar symptoms and imaging studies nor outcome.

According to the clinical score considered for the present study, more than half of patients (56%) were classified as having low, 48 patients (39%) moderate and only 7 patients (5.6%) severe AC/ACA.

The median time at onset of cerebellar symptoms was 6 days before hospitalization (range 0–30 days).

The median length of the hospital stay was 11 (range 1–29) days.

Lumbar puncture was performed in 24 children (19, 5%), showing pathological findings in 8 cases (33%). Pathological findings were pleocytosis in 8 cases (mean 51 cells/mmc, range 7–150) and elevated proteinorrachia in 4 cases (50–528 mg/l). The cerebrospinal fluid (CSF) glucose was normal in all cases. There was no correlation between CSF findings and both clinical severity and outcome (*P*> 0.05).

A definitive microbiological diagnosis was obtained in 2 cases by positive PCR on cerebrospinal fluid (VZV and parainfluenzae virus).

CT was performed in 28 cases (23%) showing pathological findings in 2 cases (cerebellar oedema in one case and bilateral hypodensity areas of the cerebellar parenchyma in the other case). Children with a severe clinical score had a higher probability of having a pathological CT compared with children with a low-moderate clinical score (*P*= 0.009).

A magnetic resonance imaging (MRI) was performed in 61 cases showing pathological results in 6 cases (5%):

- hyperintense signal of the cerebellar peduncoli in T2- weighted sequences
- hyperintensity of the cerebellar white matter in T2- weighted sequences
- Pial enhancement along the folia in 2 cases
- hyperintensity of the cerebellar grey matter in T2- weighted sequences in 2 cases

There was no correlation between clinical severity and MRI findings (*P*> 0.05).

An electroencephalogram (EEG) was performed in 45 cases (36% cases) showing abnormal findings in 20 cases (16%): Main findings slow bilateral activity in the occipital regions. There was no correlation between clinical severity and EEG findings (*P* > 0.05).

Treatment was decided independently case by case. Eighty-one patients (65%) were treated with intravenous acyclovir for 5 days.

Forty one patients (33%) received intravenous steroids. Patients with a higher clinical score on admission had a higher probability of receiving intravenous steroids (*P*=0.006).

### VZV- related AC/ACA

We separately analyzed the 66 children (53% of cases) with VZV-related AC/ACA (Table 6).

**Table 6:** Main findings of VZV and non-VZV related AC/ACA

	VZV		No-VZV		*P* Value
	N	%	N	%	
Sex (*N*=104)^a^					
Male	34	52	23	61	0.37
Female	32	48	15	39	
Age, years (*N*=104)^b^ *[median, range]*	5	1–16	4	1–17	0.17
Score (*N*=104)^c^					
Low	35	53	18	47	0.89
Moderate	27	41	18	47	
Severe	4	6	2	5	
Steroids (*N*=68)^a^					
No	12	32	17	55	0.06
Yes	25	68	14	45	
Antivirals (*N*=94)^c^					
No	3	5	12	13	0.001
Yes	55	95	24	67	
CSF findings (pathological) (*N*=19)^c^					
No	5	50	7	78	0.35
Yes	5	50	2	22	
MRI (pathological) (*N*=52)^c^					
No	28	90	19	90	1
Yes	3	10	2	10	
CT (pathological) (*N*=24)^c^					
No	10	91	12	92	1
Yes	1	9	1	8	
EEG (pathological) (*N*=37)^a^					
No	7	44	12	57	0.42
Yes	9	56	9	43	

^a^Chi^2^

^b^Mann-Whitney test

^c^Fisher exact test. *CSF* cerebrospinal fluids

VZV was diagnosed on a clinical basis since sierological tests to confirm Chickenpox are not necessary. All patients presented with cerebellar symptoms either on the crusted phase or in any case not before day 5 of the disease. Clinical presentation and outcome did not changed based on the day of the disease.

This sub-group of children have a similar rate of pathological CSF, EEG and neuroimaging findings compared to non VZV AC/ACA (*P* >0.05). Similarly, clinical severity on admission, length of stay in hospital and outcome were similar between the two groups (*P* > 0.05).

### Outcome

Median follow-up time was 1 year. Neurological sequelae were reported in 6 cases (5%) (Table 1). In particular, at a median 12 month-follow up, the following sequelae were reported: ataxia in 3 cases, balance disturbances and dysarthria in 1 case, ataxia and balance disturbances in 1 case and ataxia and hypotonia in 1 case. No cognitive function nor behavioral changes were noted.

There was no correlation between clinical manifestations and clinical score on admission and length of hospital stay, sex, age and EEG findings with sequelae (*P* > 0.05).

Children with pathological MRI had a higher probability of having clinical sequelae (*P*=0.024), even though 50% of children with pathological MRI had a complete recovery.

Similarly, a pathological CT on admission was strongly associated with neurological sequelae (*P* 0.004).

Considering the whole group of patients, outcome was not affected by antiviral and steroid therapy (*P* >0.05), despite the mode of administration and length of treatment. Nevertheless, 95% of VZV-patients received antivirals; therefore a control-group for VZV who did not receive antivirals was not available.

## Discussion

In this report we described clinical, laboratory, neuroimaging and neurophysiologic findings, treatment and outcome for 124 children admitted for AC/ACA in 12 Italian hospitals over a 10 year period.

In our case series, the median age of children affected by AC/ACA was about 4.5 years, similar to previously published reports [13–15].

The mean time between signs/symptoms at onset (fever, rash, viral infections) and onset of cerebellar symptoms was 6 days (range 0–30 days), similar to most reports describing a median time of 7 days between the onset of VZV exanthema and hospital admission at 7 days [13].

Children remained a median of 11 days in hospital. The hospitalization of our patients was longer than those reported in the literature (6.72 days) [13, 14, 16], probably due to the fact that 6 patients had AC with neuroradiologic abnormalities for which clinicians decided to treat for steroids for more than 14 days.

At admission, ataxia was the most frequent sign, with wide-based gait (92%). Neurological presentation was also often characterized by dysmetry and difficult speech. Vomiting, fever and headache were frequent non cerebellar symptoms (up to 40% of cases), while nystagmus or other involuntary eye movements were rare. In the literature one of the most detailed descriptions of the clinical presentation for VZV- related cerebellitis showed similar findings [17].

**Fig. 2:**
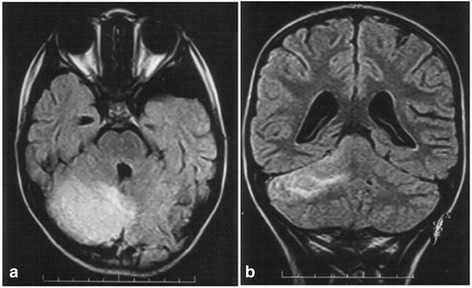
**a** Flair MRI lesion subcortical white matter right cerebellar hemisphere (case 6). **b** Flair MRI diffuse cortical lesion right cerebellar hemisphere

**Fig. 3:**
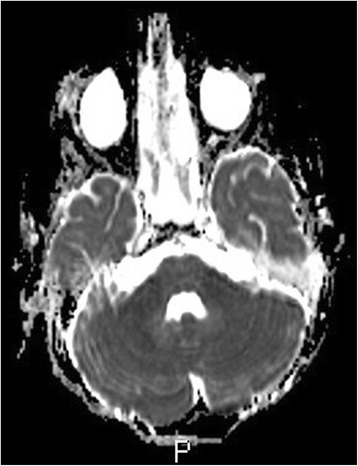
MRI brain after administration of mdc nuanced impregnation of the meninges in the cerebellar lobe in some places shows micro nodular appearance (case 4)

Brain MRI was performed in half of cases (48.4%) showing pathological results in 6 cases (10%), such as hyperintense signal of the cerebellar peduncoli in T2- weighted sequences (16%), hyperintensity of the cerebellar white matter in T2- weighted sequences (16%) (Fig. 2), pial enhancement along the folia in 2 cases (32%) (Fig. 3) and hyperintensity of the cerebellar grey matter in T2- weighted sequences in 2 cases (32%). The possibility of various patterns of cerebellar involvement in AC have been already reported [3] and our findings are similar to those described. In our series, a pathological brain MRI was not associated with more severe clinical symptoms/clinical score but was associated with a higher probability of neurological sequelae (*P*=0.024). This finding is different from the largest review of MRI findings in acute cerebellitis, which evaluated 29 cases of AC with a clinical and neuroimaging follow-up of 33 months (mean age 13.5 years, 1–64 years – 26 children < 18 years) and found poor correlation with the outcome of the patient [3].

Brain CT on the first days of admission was performed in 22% of cases showing pathological findings in 2 cases (cerebellar edema in one case and bilateral hypodensity areas of the cerebellar parenchyma in the other case). Children with a severe clinical score had a higher probability of having a pathological CT compared with children with a low-moderate clinical score (*P*= 0.009). Interestingly, a pathological CT at admission was strongly associated with neurological sequelae (*P*= 0.004). These findings may have a clinical consequence since these patients could be those selected for steroid therapy, even though currently there are no data in literature to suggest this strategy. Nevertheless, the potential utility of brain CT in the acute phase, to detect acute hydrocephalus, cerebellar edema or brainstem compression has already been proposed since a few cases of AC [3], with hydrocephalus as the presenting symptom, have been described in the literature [18–20].

It is important to note that in the majority of Italian centers it is difficult to obtain emergency brain MRI, but in most cases there is a usual “waiting time” of about 7 days, while it is easy to have a brain CT scan at the time of patient presentation in the emergency department. This delay could explain the low number of pathological MRI we found in this series and therefore some cases of AC may have been missed because of this.

Despite the frequency of AC/ACA cases in pediatric clinical practice, the role of antiviral and steroidal therapy is controversial and poorly studied [21–26]. International guidelines do not clearly establish whether immunocompetent children with cerebellitis should receive intravenous aciclovir and/or steroids, and in our case series each center decided case by case by personal experience and local guidelines, usually based on clinical severity. In our series, intravenous aciclovir was used in 81 patients (66%) despite a VZV infection being diagnosed in 66 cases, while intravenous steroids were used in 41 (33%).

In the majority of published reports on VZV related AC/ACA aciclovir is widely used [27, 13, 17, 28], as also suggested by Heininger et al. who considered the use of antivirals to be mandatory not only for patients at risk of severe disease, but also for any subject with VZV infection with virally mediated complications, such as AC/ACA [26], despite no clear benefits having ever been described.

Similarly, most authors use steroids, mainly for more complicated cases [13, 17, 28], but it is not clear what is meant by more severe cases and which is the best steroid, dosage, mode of administration and length of therapy. In our series, we found that antiviral and steroid therapy had no benefits on outcome if we consider the whole group of patients, since most cases had a good outcome with complete recovery and only 6 children (5%) had neurological sequelae, despite treatment received. Nevertheless, 95% of VZV-patients received antivirals and 68% steroids, therefore for this single group we did not have a control group who did not receive therapy. For this reason we cannot speculate for this sub-group of patients whether antivirals/steroids would benefit the outcome or not.

In any case, since we demonstrated that children with pathological brain CT or MRI on admission had a higher probability of having long-term neurological sequelae, this sub-set of patients could be the one to select for early and more aggressive treatment.

AC/ACA is the most common neurological complication of varicella occurring in about 1/4000 varicella cases among children [29–33].

In literature we found other reports which describe infection of VZV and neurological complication (encephalitis, meningitis, cerebellitis, poliradiculopaty, transverse myelitis) in adults and children [34, 30, 31]. Other reports describe Acute ataxia in Children but they analyzed a different pathogenesis, not only infectious and post infectious AC [32, 33]. However only the report cited in our work matched properly with our criteria ie AC in children with Varicella. Moreover the report of Bozzola et al. [17], described a detailed clinical presentation of AC in 48 children among 457 patients hospitalized for Chickenpox retrospectively evaluated over a period of 10 years.

In our series, a proportion of 66 children (54% of cases) received a diagnosis of varicella before the onset of AC/ACA. This subset of children did not have a different severity at presentation nor a different outcome. All children with VZV-related AC/ACA were not vaccinated, but we found 10 children who received VZV vaccination and developed non VZV-related AC/ACA.

This finding is of interest if we consider VZV immunization policies in Italy, where since 2003 only a few Italian Regions have had coverage and it is still very low in most of the other Italian Regions. In this regard, an interesting finding is that one of the major Italian pediatric Hospitals working in an area that offers free VZV vaccination with high vaccination coverage, reported only a few cases of AC/ACA. This data highlights the potential benefits of spreading VZV vaccinations since the prevention of disease may also reduce the complications of VZV infection.

In our series, 6 children presented neurological sequelae (ataxia in 3 cases, balance disturbances and dysarthria in 1 case, ataxia and balance disturbances in 1 case and ataxia and hypotonia in 1 case). The only predicting parameters significantly associated with sequelae were a pathological MRI (*P*=0.024) and/or CT on admission (*P* 0.004).

Only a few studies so far have examined the sequelae of AC/ACA mainly addressing neurological problems in the short-term period. [3, 7, 35]. Connolly et al. [35] found that up to 91% of children with AC/ACA had complete neurological recovery after 4 months. It is of note that this study included mainly children without mental status changes and normal imaging studies.

On the contrary, Hennes et al. described a series of of 11 children (age range: 3 years to 14 years and 10 months), six of them with a severe disease manifestation including mental status changes MRI abnormalities, which were followed up over a mean period of 4 years and 4 months. [8] Neurological sequelae were reported in five children ranging from ataxia to mild tremor, and cognitive deficits (spatial visualization ability, language skills, and concentration) were found in six patients. This report might confirm our findings which suggested that a baseline pathological brain MRI/CT is associated with neurological sequelae, therefore suggesting a potential subgroup which might benefit from more aggressive treatment. Obviously, these findings need to be confirmed by prospective studies before advising routinely perform ED baseline neuroimaging studies on all children presenting with AC/ACA. Studies exploring clinical data able to predict neuroimaging abnormalities could be useful in this selection. Unfortunately in our study we did not find any clinical presentation associated with abnormal neuroimaging studies. The main limits of this study are the retrospective nature and the lack of neuroimaging follow-up for those with baseline pathological CT-MRI. Nevertheless, this is the largest series of pediatric AC/ACA reporting a detailed clinical, laboratory, neuroimaging, electrophysiological picture of this condition and analyzing how these findings and treatment options correlate with outcome.

## Conclusions

We conducted this study to define the clinical, neuroimaging and electrophysiologic features of patients with AC/ACA and to evaluate the association of their clinical manifestations and the therapeutic strategies with the neurological outcome of the disease. We confirmed the literature data about the benign course of AC/ACA in most cases but we also highlighted a considerable rate of patients with neurological sequelae (5%). So we analyzed all the data in order to find the risk factors and the early predictive signs of adverse outcome but such evaluation did not show a correlation between sequelae and clinical manifestations or therapeutic strategies, or CSF findings. The only statistically significant association was found between pathological MRI or CT images at admission and the neurological sequelae, in addition to a relationship between the severity of clinical manifestations and a pathological CT. These findings could suggest indications to perform a neuroimaging evaluation for all patients with AC/ACA at admission to identify those at higher risk of neurological outcome that might benefit from a more aggressive therapeutic strategy and should have a closer follow-up. However, we cannot indicate routinely performing such instrumental evaluation in patients with AC/ACA only on the basis of a retrospective study. Therefore, we believe that randomized controlled trials are needed to better define the relationship between clinical manifestation, therapy and neurological outcomes and to confirm our data on the relevance of MRI and/or CT at diagnosis of the disease. The ultimate goal of these studies could be to develop a standardized protocol about the neuroimaging approach in the evaluation of AC/ACA that is now defined by the independent decision of each center. The MRI/CT data, associated with the clinical manifestations, might allow us to define the class risk of patients for neurological outcomes in order to standardize not only therapeutical strategies but also the follow-up program.

## Abbreviations

AC: Acute cerebellitis
ACA: Acute cerebellar ataxia
CSF: Cerebrospinal fluid
CT: Computed tomography
EEG: Electroencephalogram
HHV-6: Human Herpes Virus 6
MRI: Magnetic resonance imaging
VZV: Varicella Zoster Virus
